# The performance of the World Rugby Head Injury Assessment Screening Tool: a diagnostic accuracy study

**DOI:** 10.1186/s40798-019-0231-y

**Published:** 2020-01-09

**Authors:** G. W. Fuller, R. Tucker, L. Starling, E. Falvey, M. Douglas, M. Raftery

**Affiliations:** 1 Centre for Urgent and Emergency Care Research, School of Health and Related Research, University of Sheffield, Regent Court, 30 Regent Street, Sheffield, S1 4DA UK; 2 University of Cape Town School of Management Studies, Cape Town, South Africa; 3 Department of Human Biology, University of Cape Town, Cape Town, South Africa; 4 Department of Medicine, University College Cork, Cork, Ireland; 5 World Rugby, World Rugby House 8-10 Lower Pembroke Street, Dublin, Dublin 2 Ireland

**Keywords:** Concussion, Screening, Rugby, Diagnostic accuracy

## Abstract

**Background:**

Off-field screening tools, such as the Sports Concussion Assessment Tool (SCAT), have been recommended to identify possible concussion following a head impact where the consequences are unclear. However, real-life performance, and diagnostic accuracy of constituent sub-tests, have not been well characterized.

**Methods:**

A retrospective cohort study was performed in elite Rugby Union competitions between September 2015 and June 2018. The study population comprised consecutive players identified with a head impact event undergoing off-field assessments with the World Rugby Head Injury Assessment (HIA01) screening tool, an abridged version of the SCAT3. Off-field screening performance was investigated by evaluating real-life removal-from-play outcomes and determining the theoretical diagnostic accuracy of the HIA01 tool, and individual sub-tests, if player-specific baseline or normative sub-test thresholds were strictly applied. The reference standard was clinically diagnosed concussion determined by serial medical assessments.

**Results:**

One thousand one hundred eighteen head impacts events requiring off-field assessments were identified, resulting in 448 concussions. Real-life removal-from-play decisions demonstrated a sensitivity of 76.8% (95% CI 72.6–80.6) and a specificity of 86.6% (95% CI 83.7–89.1) for concussion (AUROC 0.82, 95% CI 0.79–0.84). Theoretical HIA01 tool performance worsened if pre-season baseline values (sensitivity 89.6%, specificity 33.9%, AUROC 0.62, *p* < 0.01) or normative thresholds (sensitivity 80.4%, specificity 69.0%, AUROC 0.75, *p* < 0.01) were strictly applied. Symptoms and clinical signs were the HIA01 screening tool sub-tests most predictive for concussion; with immediate memory and tandem gait providing little additional diagnostic value.

**Conclusions:**

These findings support expert recommendations that clinical judgement should be used in the assessment of athletes following head impact events. Substitution of the tandem gait and 5-word immediate memory sub-tests with alternative modes could potentially improve screening tool performance.

## Keypoints

- The World Rugby Head Injury Assessment process demonstrated good performance for the identification of concussion.
- Clinical judgement was commonly used to interpret off-field concussion screening tool results, resulting in improved performance compared to the strict application of baseline or normative test thresholds.
- Symptoms and clinical signs were the HIA01 screening tool sub-tests most predictive for concussion; substitution of the tandem gait and 5-word immediate memory sub-tests with alternative modes could potentially improve screening tool performance.

## Background

Concussion remains a common and high profile injury in contact and collision sports, with an incidence ranging between 15 and 20 concussions per 1000 player-match-hours in professional Rugby Union. Continuing to play with a concussion is associated with increased risk of further head or non-head injury, worsened severity and delayed recovery [1].

Elite sports have consequently introduced processes to identify and manage head impact events during matches [2]. These procedures typically involve immediate removal of players with clearly apparent signs of concussion (e.g. loss of consciousness). Where the circumstances or consequences of a head impact are unclear (e.g. dangerous mechanism), brief off-field screening tests are commonly used to identify possible concussion, with the diagnosis subsequently confirmed or refuted by detailed clinical assessments conducted in the hours and days following the head impact event [2–4]. World Rugby’s concussion management system is the Head Injury Assessment (HIA) process.

The 5th Berlin Consensus statement on Concussion in Sport recommends a multi-modal screening approach, incorporating multiple sub-tests, as exemplified by the Sports Concussion Assessment Tool (SCAT) [4, 5]. Sub-test thresholds indicating abnormal SCAT performance are not explicitly defined, and athlete-specific pre-season baseline values, or population normative ranges, are commonly used to aid interpretation of results. The Berlin consensus statement highlights that ‘individual management and return-to-play decisions remain in the realm of clinical judgement’ [4].

Despite the prominence of sports-related concussion, a recent systematic review concluded that the performance of existing off-field concussion screening tools and sub-tests has been poorly characterised [3]. The aim of the current study was therefore to investigate the performance of the World Rugby HIA01 off-field concussion screening tool, an abridged version of the SCAT3, in professional Rugby Union. Specific objectives were to characterise the performance of real-life return to play decisions; evaluate the theoretical diagnostic accuracy of the HIA01 screening tool if baseline or normative sub-test thresholds were strictly applied; and quantify the diagnostic accuracy and relative contribution of individual HIA01 screening tool constituent sub-tests.

## Methods

### World Rugby HIA process

The 3-stage HIA process has been described in detail previously [6, 7]. Briefly, players enter stage 1 of the HIA process following the identification of a meaningful head impact event during a game, either by direct observation or video review. Players demonstrating clear signs of concussion (termed ‘Criteria 1’, e.g. loss of consciousness, tonic posturing, ataxia) are immediately and permanently removed from the match, without undergoing off-field concussion screening. Where the circumstances or consequences of a head impact event are not clear (termed ‘Criteria 2’, e.g. dangerous head impact event mechanism), players undergo off-field screening for a possible concussion, with a return to play only if the assessment is judged to be normal. All players subsequently undergo detailed medical assessments by the team doctor within 3 h of the head impact event (HIA02 assessment), and after 2 nights rest (HIA03 assessment), to monitor clinical progress and to confirm or refute a diagnosis of concussion.

### Study design, setting and study population

A retrospective diagnostic accuracy cohort study was performed using prospectively collected data from the World Rugby Head Injury Assessment (HIA) database. The source population comprised elite rugby players participating in elite-level International and national competitions across the world between September 2015 and June 2018. This period was chosen as no important operational changes were made to World Rugby HIA processes over this time. The subsequent study population included all players identified during play with a meaningful head impact event, but unclear consequences, undergoing off-field screening assessment for a possible concussion. Players with overt Criteria 1 signs of concussion were not considered in the study population as these cases are immediately and permanently removed from play without off-field concussion screening. The final study sample was composed of players for whom a final diagnosis was known.

### Index tests and reference standard

The index test under investigation was the World Rugby HIA01 off-field screening assessment, consisting of the HIA01 screening tool administered by the team doctor or match-day doctor [6]. The assessment is conducted in an off-field medical room during a 10-min player interchange. The HIA01 screening tool, an abridged version of the SCAT3 [8], comprises 6 sub-tests: Maddock’s questions, tandem gait test, immediate and delayed recall, digits backwards, and an abbreviated symptom checklist. An evaluation of clinical signs is also conducted during the off-field assessment for any other suggestion of possible concussion (e.g. irritability, poor concentration, drowsiness).

Performance in the sub-tests is interpreted in comparison to previously collected baseline values, or if absent, a normative cut-point derived from a large sample of professional Rugby Union players [9]. If HIA01 off-field screening test performance is considered to be normal a player is cleared for return to play, with no concussion concerns. Sub-test results judged to be abnormal indicate a possible concussion, leading to permanent removal of the player from the remainder of the game. It is also possible to remove a player from further match participation, despite the otherwise normal sub-test performance, if clinical signs of possible concussion are observed, e.g. emotional, poor concentration. HIA01 screening tool content and normative cut-points are summarised in Table 1.

**Table 1:** Constituent sub-tests in the HIA01 off-field screening tool

Sub-test	Domain	Description	Score range^a^	Threshold for abnormality
Maddock’s questions	Orientation	•What venue are we at today? •Which half is it now? •Who scored last in this match? •What team did you play last week/game? •Did your team win the last game?	0–5	< 5
Immediate memory	Cognition	Remembering a list of 5 words in 3 trials	0–15	< 12 or less than baseline
Digits backwards	Cognition	Repeating word strings (increasing from 3- to 6-word lengths) in reverse order	0–4	< 3 or less than baseline
Tandem gait	Balance	6 m line heel-to-toe gait along a straight line	Continuous	> 14 s
Symptom checklist	Symptoms	•Do you have a headache? •Do you have any dizziness? •Do you have any ‘pressure in your head’? •Do you feel nauseated or do you feel like vomiting? •Do you have any blurred vision? •Does the light or noise worry you? •Do you feel as though you are slowing down? •Do you feel like you are ‘in a fog’? •Do you feel unwell?	0–9	> 0
Delayed Recall	Cognition	Remembering previous list of 5 words in any order	0–5	< 3 or less than baseline
Clinical signs	Subjective signs of a possible concussion	•Emotional - sad, anxious, nervous, irritable •Drowsy •Difficulty concentrating •Doctor suspects possible concussion for other reasons.	0–1	> 0

^a^High score represents better performance for all tests except symptoms and clinical signs

The reference standard, against which performance was compared, was the team doctor’s diagnosis of concussion formulated during the HIA02 and HIA03 assessments over the 48 h post-head impact. The HIA02 assessment consists of a detailed clinical evaluation including the SCAT3 instrument. The HIA03 assessment comprises a clinical evaluation, supported by an expanded SCAT3 symptom checklist, a cognitive assessment (typically a computerised neuro-cognitive tool such as CogSport) and a balanced assessment using the balance error scoring system [10, 11]. All players entering the HIA process are subject to this clinical follow up regardless of the results of the HIA01 screening test.

### Data collection

HIA process data are routinely recorded at the point of assessment by assessing physicians using the tablet-based, web-hosted, CSx data platform [12]. Data is subsequently uploaded to the World Rugby HIA database. In the rare event that web access is unavailable assessments are recorded on paper, submitted centrally, and uploaded manually to the World Rugby HIA database. HIA assessment forms, from each of the 3 HIA process stages, are linked deterministically using unique player identifiers. Competition coordinators are responsible for data quality and collection of outstanding information.

### Analyses

The HIA process and study population characteristics were initially examined using descriptive statistics. Four aspects of the HIA01 off-field concussion screening assessment were then evaluated. First, the performance of real-life removal-from-play decisions was examined. Each case was coded according to the action taken by the clinician after the HIA01 off-field screening assessment (player removed from play with possible concussion v returned to play having been cleared of concussion). This off-field screen outcome was compared to the reference standard result (final diagnosis of concussed vs not-concussed supported by the HIA02 and HIA03 assessments). Prevalence of concussion, sensitivity and specificity, and area under the receiver operating characteristic curve (AUROC) with their 95% confidence intervals (CI), were subsequently calculated.

Second, the relationship between actual removal-from-play decisions and recorded screening tool sub-test results was characterised descriptively. Consistent with the Berlin consensus statement [13], clinical judgement could be used to interpret sub-test results in comparison to baseline values, or normative thresholds if no baseline data were available. It was therefore possible to return a player to the competition if worse performance than baseline was recorded if this was not felt to represent possible concussion, e.g. a small increase in tandem gait time, or presence of a symptom judged not to represent possible concussion. Conversely, players with normal sub-test performance could be removed if a concussion was otherwise felt to be possible based on the detection of other clinical signs during the off-field assessment (e.g. emotional, drowsy).

Third, the theoretical performance of the HIA01 screening tool was investigated by analysing the sub-test results assuming that test thresholds were strictly applied, rather than interpreted as usual with clinical judgement. Player-specific baseline, and population-based normative [9], sub-test thresholds were examined separately. The index test was classified as normal (no sub-tests results worse than player-specific pre-season baseline value or normative test thresholds) or abnormal (any sub-test result worse than preseason baseline value or normative threshold). Sensitivity, specificity and AUROC were then calculated [14]. Relative performance between real-life removal-from-play decisions and theoretical HIA01 screening tool results using strict baseline/normative thresholds was then evaluated using McNemar’s test and by comparing AUROC using de Long’s method [15, 16].

Finally, the utility of constituent HIA01 screening tool sub-components was investigated separately using baseline values and normative test thresholds. Diagnostic accuracy of each sub-test was examined through the calculation of individual sensitivities, specificities, and AUROCs. The predictive ability of individual sub-tests to identify concussion was then explored using multivariable logistic regression modelling [17]. Diagnosed concussion was the dependent variable. HIA01 off-field screening sub-tests were the independent variables, categorised as binary pass/fail variables according to the relevant baseline/normative threshold. Backwards stepwise regression was performed, with the elimination of sub-tests providing no statistically significant improvement (*p* = 0.2) in prediction of concussion. Consensus recommendations for best practice in prognostic logistic regression modelling were followed [17–19].

### Sample size, statistics and ethics

As a secondary analysis of a census sample from an existing data-set was performed, a power analysis is superfluous and the 95% confidence intervals around the effect estimate indicate the precision of results [20]. For logistic regression modelling, 10 events per variable is considered acceptable to achieve stable models [21]. The 448 concussion cases in the study sample would therefore allow examination of up to 44 variables, well in excess of those investigated. Statistical analyses were carried out in Stata version 13.1 (StataCorp, College Station, USA) with a conventional significance level (α) of 0.05 used. The following Stata add-in modules were used: *tuples*, *diagt*, *roctab*, *roccomp* and *stepwise.* Ethical approval was confirmed with the University of Sheffield. All players provided written informed consent for the use of HIA data for research purposes. All data were anonymised. The study was performed in accordance with the standards of ethics outlined in the Declaration of Helsinki.

## Results

### Derivation and characteristics of study participants

**Fig. 1:**
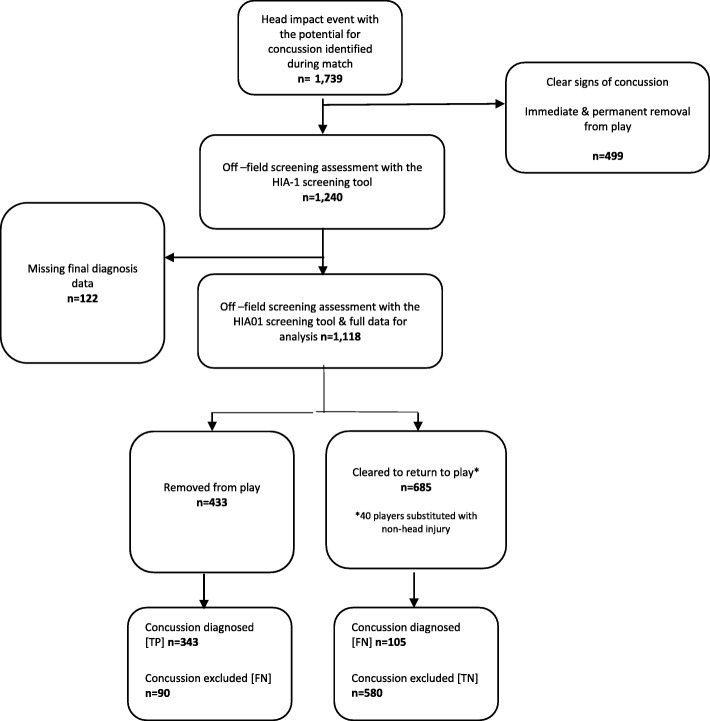
Derivation of the study sample. TP, true positive; FN, false negative; FP, false positive; TN, true negative.

A total of 1739 meaningful head impact events were detected in 1265 individual players (recurrent events occurring in 325 players, ranging from 223 players with 2, to 1 player with 10 incidents) over the study period. Of these, 499 incidents (in 450 players) were associated with overt signs or symptoms of concussion requiring immediate and permanent removal from play. The remaining 1240 incidents (in 980 players), where the circumstances and/or consequences were unclear underwent off-field concussion screening assessments, comprised the study population. Reference standard data was missing secondary to incompletely recorded follow up in 122 incidents (9.8%), leaving a study sample of 1118 players. The HIA01 off-field screen was performed by the team doctor in 706 (63.3%) and by the match day doctor in 412 (36.7%) incidents. In 433 incidents, players were removed from play following an abnormal off-field screen indicating a possible concussion. The remaining 685 players were cleared to return to play with no indication of a possible concussion. Of these cleared players, 40 players were substituted for tactical reasons, or other non-head injury, at the time of the off-field screening assessment. Figure 1 presents a flow chart describing the derivation of study participants.

### Analyses

#### Real-life HIA01 screening assessment outcomes (i.e. real-life removal-from-play decisions)

In the study sample of players undergoing off-field concussion screening, 448 head impact events had a confirmed final clinical diagnosis of concussion, giving a prevalence of 40.7% (95% CI 37.2–43.0, *n* = 1118). Of concussed players, 343 were removed from play after the HIA01 off-field screen resulting in a sensitivity of 76.6% (95% CI 72.4–80.4). Six hundred seventy players were reference standard negative with no confirmed concussion. Of these, 580 cases were cleared to return to play after the off-field screen (of whom 40 were substituted for another reason). The specificity to correctly identify players without concussion in this study group was therefore 86.6% (95% CI 83.7–89.1). The positive and negative predictive values of removal-from-play decisions were 79.2% (95% CI 75.1–83.0) and 84.8% (81.8–87.3) respectively. Diagnostic accuracy metrics are summarised in Table 2.

**Table 2:** Comparison of removal- from-play and theoretical HIA01 screening tool performance

Index test	TP	FN	FP	TN	Sensitivity (%, 95% CI)	Specificity (%, 95% CI)	AUROC (95% CI)
Real-life removal- from-play decisions^a^	343	105	90	580	76.6 (72.4–80.4)	86.6 (83.7–89.1)	0.8 (0.8–0.8)
HIA01 screening tool^b^ (baseline thresholds)	389	45	408	209	89.6 (86.4–92.3)	33.9 (30.1–37.8)	0.6 (0.6–0.6)
HIA01 screening tool^c^ (normative thresholds)	360	88	208	462	80.4 (76.4–83.9)	69.0 (65.3–72.4)	0.8 (0.7–0.8)

*TP* true positive, *FN* false negative, *FP* false positive, *TN* true negative, *AUROC* area under the curve of the receiver operating characteristic

^a^Index test: removed from play v returned to play

^b^Index test: any sub-test score worse than baseline value v no sub-test score worse than baseline value and no subjective clinical signs of a possible concussion

^c^Index test: any sub-test score worse than normative threshold v no sub-test score worse than a normative threshold and no subjective clinical signs of a possible concussion

#### Concordance of removal-from-play decisions with objective sub-test results

Player-specific baseline data were available for comparison at the time of off-field assessments for 94.0% of cases (*n* = 1051/1118). The remaining 6.0% of players (*n* = 67/1118) underwent off-field screening assessments informed by normative thresholds.

Of the cases with baseline data available, 60.2% were returned to play (*n* = 633/1051), of which 61.1% (*n* = 387/633) had at least 1 sub-test recorded where the result was worse than the player-specific baseline value. This was most frequently a single subtest (68.7%, *n* = 266/387), increasing to 4 subtests in a small minority of cases (0.5%, *n* = 3/387 cases). Tandem gait was the most common sub-test where a performance worse than baseline was evident, with a median screening result 2.55 s slower than the baseline value (IQR 1.2–6.3 s, 69.8% with the worse sub-test result, *n* = 270/387). For players assessed using player-specific threshold data and removed from play (*n* = 418), all sub-tests results were better than baseline values in 6.7% of cases (*n* = 28/418), with these players removed secondary to clinical signs of a possible concussion.

**Fig. 2:**
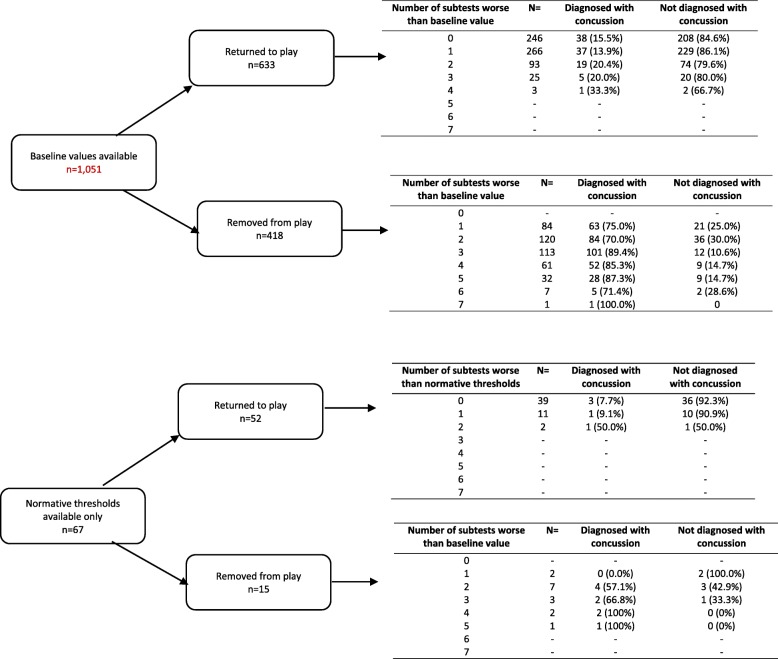
Relationship between removal-from-play decisions, objective sub-test results and final diagnosis of concussion

Of the 67 cases using normative thresholds in off-field screening assessments, 77.6% (*n* = 52/67) were returned to play. Of these, 25% (*n* = 13/52) had one or more sub-test results worse than the relevant normative thresholds. This was usually a single subtest (92.3%, *n* = 12/13), most commonly digits backwards (53.8%, *n* = 7/13). A single player was removed secondary to clinical signs of a possible concussion, despite otherwise normal sub-test performance against normative thresholds. Figure 2 summarises the concordance between removal-from-play decisions, objective sub-test results and final diagnosis of concussion. A detailed description of subtest findings is presented in the web appendix.

#### Theoretical diagnostic accuracy of HIA01 screening tool if baseline thresholds strictly applied

If individual baseline thresholds were strictly applied across the sample, with any worsening in recorded sub-test results compared to the preseason value classified as abnormal, the theoretical sensitivity of the HIA01 screening tool would have been 89.6% (95% CI 86.4–92.3), with a specificity of 33.9% (95% CI 301.1–37.8). This sensitivity was significantly higher, and specificity significantly lower, than the performance of the off-field screen observed with actual removal- from-play decisions (McNemar’s test, *p* < 0.01, *n* = 1051). Sensitivity did not significantly differ from removal- from-play decision performance if normative thresholds were strictly applied to all cases (80.4%, 95% CI 76.4–83.9, *p* = 0.41), but specificity was significantly worse at 69.1% (95% CI 65.5–72.6, *p* < 0.01, *n* = 1118). The overall discriminatory ability of strictly applying baseline or normative thresholds (AUROC 0.6 and 0.8 respectively) was worse than that achieved when the clinical judgement was used to guide actual return to play decisions (*p* < 0.001). Diagnostic accuracy metrics for the HIA01 screening tool are summarised in Table 2.

#### Diagnostic utility of individual HIA01 sub-tests

The individual diagnostic accuracy of constituent sub-tests of the HIA01 off-field screening tool, classified according to baseline and normative thresholds, are presented in Tables 3 and 4 respectively. Sensitivity was individually low for all sub-tests, ranging from 1.8% for the Tandem Gait to 55.6% for the symptom checklist (both when applying normative thresholds). Specificities were generally higher, with estimates ranging from 56.6 to 99.7% for the tandem gait sub-test using baseline and normative thresholds respectively. Using normative thresholds, rather than player-specific baseline values, resulted in significantly lower sensitivities, but improved specificity for each test, with the exception of Maddocks questions and the symptom checklist (*p* < 0.01).

**Table 3:** Diagnostic accuracy of HIA01 screening tool sub-tests compared if baseline thresholds applied^a^

Sub-test	TP	FN	FP	TN	Sensitivity (%, 95% CI)	Specificity (%, 95% CI)	AUROC (95% CI)
Maddocks questions	36	398	13	604	8.3 (5.9–11.3)	97.9 (96.4–98.9)	0.5 (0.5–0.6)
Immediate memory	75	359	71	546	17.3 (13.8–21.2)	88.5 (85.7–90.0)	0.5 (0.5–0.6)
Digits backwards	145	289	93	524	33.4 (29.0–38.1)	84.9 (81.9–87.7)	0.6 (0.6–0.6)
Tandem gait	203	231	268	349	46.8 (42.0–51.6)	56.6 (52.5–60.5)	0.5 (0.5–0.6)
Symptoms	211	223	50	567	48.6 (43.8–53.4)	91.6 (89.5–93.9)	0.76 (0.7–0.7)
Delayed recall	158	276	115	502	36.4 (31.9–41.1)	81.4 (78.1–84.4)	0.6 (0.6–0.66)
Clinical signs^b^	189	259	43	627	42.2 (37.6–46.9)	93.6 (91.5–95.3)	0.7 (0.7–0.7)

*TP* true positive, *FN* false negative, *FP* false positive, *TN* true negative, *AUROC* area under the curve of the receiver operating characteristic

^a^Baseline threshold = any worsening in off-field screening sub-test performance compared to previous baseline value

^b^Sub-test does not have normative or baseline threshold

**Table 4:** Diagnostic accuracy of HIA01 screening tool sub-tests compared if normative thresholds applied^a^

Sub-test	TP	FN	FP	TN	Sensitivity (%, 95% CI)	Specificity (%, 95% CI)	AUROC (95% CI)
Maddocks questions	40	408	16	654	8.9 (6.5–12.0)	97.6 (96.2–98.6)	0.5 (0.5–0.6)
Immediate memory	22	426	9	661	4.9 (3.1–7.3)	98.7 (97.5–99.4)	0.5 (0.5–0.5)
Digits backwards	152	296	106	564	33.9 (29.6–38.5)	84. (81.2–86.9)	0.6 (0.6–0.6)
Tandem gait	8	440	2	668	1.79 (0.78–3.49)	99.7 (98.9–100.0)	0.5 (0.5–0.5)
Symptoms	249	199	71	599	55.6 (50.8–60.2)	89.4 (86.8–91.6)	0.7 (0.7–0.8)
Delayed recall	89	359	34	636	19.9 (16.3–23.9)	94.9 (93.0–96.5)	0.6 (0.5–0.6)
Clinical signs^b^	189	259	43	627	42.2 (37.6–46.9)	93.6 (91.5–95.3)	0.7 (0.7–0.7)

*TP* true positive, *FN* false negative, *FP* false positive, *TN* true negative, *AUROC* area under the curve of the receiver operating characteristic

^a^Normative threshold = off-field screening sub-test performance worse than the normative threshold detailed in Table 1.

^b^Sub-test does not have normative or baseline threshold

Stepwise, backwards elimination, logistic regression demonstrated that immediate memory and tandem gait were not significant predictors of concussion when using either normative or baseline thresholds (both variables excluded from the model at the *p* = 0.2 level). Maddock’s questions were a weak predictor of concussion (excluded from the normative threshold model; odds ratio 1.9 95% CI 0.9–4.3 in baseline threshold model). Symptoms and clinical signs of possible concussion were the strongest predictors of concussion. Model odds ratios are presented in Table 5. Model checking and internal validation were satisfactory.

**Table 5:** Sub-tests predicting concussion, logistic regression model odds ratios

Sub-test	Odds Ratio	Standard error	z	*p* value	95% LCL	95% UCL
Baseline thresholds						
Maddocks questions	1.9	0.8	1.6	0.1	0.9	4.2
Immediate memory^a^	–	–	–	–	–	–
Digits backwards	1.9	0.4	3.5	< 0.01	1.3	2.7
Tandem gait^a^	–	–	–	–	–	–
Symptoms	6.8	1.3	10.1	< 0.01	4.7	9.9
Delayed recall	1.8	0.3	3.4	< 0.01	1.3	2.5
Clinical signs	6.9	1.6	8.6	< 0.01	4.5	10.8
Normative thresholds						
Maddocks questions^a^	–	–	–	–	–	–
Immediate memory^a^	–	–	–	–	–	–
Digits backwards	1.9	0.3	3.7	< 0.01	1.4	2.7
Tandem gait^a^	–	–	–	–	–	–
Symptoms	6.8	1.2	11.2	< 0.01	4.9	9.5
Delayed recall	3.7	0.9	5.3	< 0.01	2.3	6.1
Clinical signs	6.6	1.5	8.6	< 0.01	4.3	10.1
	–	–	–	–	–	–

*LCL* lower confidene limit, *UCL* upper confidence limit

^a^Excluded from final prediction model

## Discussion

### Summary of results

Removal- from-play decisions following World Rugby HIA01 off-field screening assessments had moderate diagnostic accuracy for the identifying concussion, demonstrating a sensitivity of 76.8% (95% CI 72.6–80.6), and a specificity of 86.6% (95% CI 83.7–89.1, AUROC 0.8, 95% CI 0.8–0.8). Clinical judgement was commonly used to interpret the off-field screening tool results since the majority of players returned to play had at least one objective sub-test result that was worse than the relevant individual baseline value or normative threshold (58.5%, *n* = 400/684). A smaller proportion of players were removed from play with clinical signs of possible concussion despite otherwise normal sub-test results compared to baseline/normative thresholds (6.7%, *n* = 29/433).

In the event that sub-test results had been acted upon strictly according to baseline values/normative thresholds, and not interpreted with additional clinical judgement, theoretical HIA01 screening tool performance worsened for both baseline (sensitivity 89.6%, specificity 33.9%, AUROC 0.6, *p* < 0.01) and normative (sensitivity 80.4%, specificity 69.0%, AUROC 0.8), *p* < 0.01) thresholds. Clinical signs and the symptoms checklist had the best sub-test performance. Tandem gait, immediate recall and Maddocks’s questions had low sensitivity for concussion and provided minimal or no diagnostic gain beyond symptoms, digits backwards, delayed recall and clinical signs for the identification of concussion cases.

### Interpretation of results

It is essential to note that following the identification of a meaningful head impact event during play, stage 1 of HIA process consists of 2 components [6]. Players demonstrating obvious signs of concussion are immediately and permanently removed from the match (Criteria 1). Off-field screening with the HIA01 tool is only performed for incidents where the consequences of a head impact event are unclear. If both components of stage 1 of the HIA process are considered together, with players immediately removed with clear signs of head injury included, then 89% of confirmed concussion cases with match-day detected head impact events were correctly identified and removed from play. Further, 13% of players without concussion were incorrectly removed from play. The performance of the complete HIA01 stage 1 process (sensitivity 90%, specificity 87%) therefore appears comparable to other diagnostic modalities used in sports medicine, e.g. MRI for rotator cuff tears (sensitivity 90%, specificity 90%) [22]. Further improvements to the HIA process will need to target the small minority of concussion cases currently ‘missed’ by the off-field screen; and improvements in diagnostic accuracy are therefore likely to be challenging.

Clinical judgement has been shown to be important in diagnosis across all fields of medicine and has been frequently demonstrated to outperform strict adherence to clinical decision rules [23, 24]. It is therefore not surprising that additional clinical judgement was applied to interpret subtest results ostensibly worse than baseline values or normative thresholds. Real-life return to play decisions using clinical judgement and clinical suspicion had improved overall discrimination for concussion compared to the strict application of baseline or normative thresholds. This supports the Berlin consensus document’s statement that the final determination of a screening assessment is a ‘medical decision based on clinical judgement’ [2, 4]*.*

Real-life return to play decisions demonstrated markedly improved specificity compared to the strict application of baseline or normative sub-test thresholds. However, this was achieved at the cost of a small reduction in sensitivity. False-negative and false-positive diagnoses are rarely equally important, and the trade-off between sensitivity and specificity may vary according to different perspectives. Ideally, in order to optimize screening tool performance, clinical costs and values would be accounted for to achieve the best balance of sensitivity and specificity. Metrics such as the weighted comparison (WC) index or net benefit would allow such calculations [25]. World Rugby is planning discrete choice experiments to establish appropriate values to facilitate this approach.

The predominant discrepancies between return to play decisions and subtest results were worse performance compared to the baseline value in a single subtest, typically a slightly slower tandem gait time than baseline. As the number of subtest with worse performance against baseline increased, the prevalence of concussion was also higher. A possible application of this finding could therefore be to enforce removal from play in the presence of multiple sub-tests results worse than baseline or normative thresholds. However, there were few cases where this occurred, suggesting that this approach would only slightly reduce the number of false negatives, with a concurrent increase in false positives.

The finding that tandem gait and 5-word immediate recall added limited or no additional diagnostic gain suggests these sub-tests could be omitted or replaced in an abridged HIA01 off-field screening test without loss of diagnostic accuracy. As additional sub-tests are added the overall sensitivity of a screening assessment will tend to increase (i.e. the number of false-negative cases falls), but specificity will generally decrease (i.e. the number of false positives rises) [25]. Adding new sub-tests to the HIA01 off-field screening tool, either from the SCAT5, or other novel side-line modalities, could therefore reduce the number of false-negative cases. World Rugby is prospectively investigating both of these approaches currently.

Including clinical signs of possible concussion as part of the HIA01 off-field screening assessment increased the detection of concussion cases. It was not possible to fully explore the reasons underlying these decisions from the current data, but possibilities could include observation of clinical deficits not currently tested in the screening tool, consideration of sub-perfect global performance not meeting test thresholds, or an overall intangible clinical impression. Further qualitative research would be helpful to explore this and could reveal targets for new screening tests. It is unlikely that clinical suspicion, within the HIA01 screening tool, operates independently of constituent sub-tests and its value could change if the screening tool were modified, and the opportunity to form a global assessment alters.

These data may suggest the limited utility of baseline test thresholds. Although screening tool sensitivity significantly improved when strictly applying player-specific baseline values, a drastic trade-off in reduced specificity was observed. Although slightly different in content from the HIA01 screening tool, relatively high intra-athlete variability has been reported previously for serial SCAT assessments [26, 27]. Worse results than baseline would therefore be frequently expected secondary to natural variation in test performance. However, baseline testing may provide additional general information to guide interpretation of individual screening tool results and may be useful for diagnosing concussion or tracking recovery. Further research could usefully investigate the effect of pre- and post-exercise baseline thresholds, and single versus repeated preseason baselines.

Alternative processes for concussion management are used in other professional sports. Off-field screening may be performed on all players following a meaningful head impact event, regardless of whether clear signs of concussion are initially present [2]. In this situation, the increased prevalence of concussion in players undergoing screening tests will have important implications for clinical decision making. All other things being equal, the negative predictive value of any screening tool will naturally fall, resulting in a reduced probability that a player does not have a concussion after a negative test [28, 29]. Moreover, ‘spectrum effects’ are likely, where the inherent diagnostic accuracy of a screening test varies according to the underlying concussion prevalence and injury severity. As more severely injured players are tested it might be expected that sensitivity would rise, and specificity would fall; but it is highly unlikely that screening test performance and negative predictive values under these circumstances would match those achieved if players with clear signs of concussion are immediately removed and permanently removed from play.

Another approach within some sports has been to use Maddocks’ questions (often on-field) initially, with further off-field testing only performed if the first test is abnormal [2]. The high false-negative rate of Maddocks questions reported in the current study suggests that this may not be safe, potentially missing up to 9 out of 10 concussions. Moreover, sequential testing, where a subsequent test is performed only if the result of a previous screening test was positive, tends to improve specificity at the cost of worsened sensitivity [30]. This could be considered sub-optimal for a high profile injury such as concussion, where minimizing false negatives is likely to be paramount, and current screening tools appear to have good specificity already.

The source population across the top tiers of the professional club and international Rugby should ensure that these results are generalisable throughout elite Rugby Union competitions. External validity to the elite level of other sports with different frameworks for evaluating head impact events is less certain. Context-specific factors that could influence the performance of sub-tests and clinical suspicion include the setting of testing (on-field v pitch-side v medical room) or equipment (boots v skates). Finally, generalisability could be reduced following the introduction of a new SCAT version, with an increase in the number of words in the immediate memory test from 5 to 10 words, potentially improving the performance of this sub-test, by removing the ceiling effects [5].

### Comparison with previous studies

The operational performance of the World Rugby HIA01 off-field screening tool has been previously investigated in a smaller 2017 Rugby World Cup sample, with similar results reported for the prevalence of concussion, sensitivity and specificity [6]. A previous iteration of the WR screening tool, the Pitchside Concussion Assessment (PSCA) tool, has also been investigated, demonstrating a sensitivity of 85% and specificity of 74%. Comparison with the current results is limited by the different screening tool content (Maddocks’ Score, tandem gait, symptom checklist, mental status checklist), administration (5 min duration) and study population (fewer immediate removals with clear signs of concussion) [7]. The prevalence of concussion has also increased since this study was performed, suggesting a lowering of the diagnostic threshold as a factor.

A recent systematic review examining the accuracy of off-field screening tools, and constituent sub-tests, reported imprecise and heterogeneous diagnostic accuracy metrics for all types of assessments [3]. Overall findings were broadly consistent with the current study, with symptoms and balance tests demonstrating high and low sensitivity respectively; and increased diagnostic gain from a combination of sub-tests into multi-modal screening tools. However, further interpretation is limited by concerns regarding clinical diversity and internal validity of individual studies.

### Limitations

This study provides a large-scale, real-life, evaluation of off-field concussion screening and has a number of strengths. Consecutive players were recruited following meaningful head impact events avoiding the bias inherent in a diagnostic case-control study designs commonly used in previous studies [31]. The index tests and reference standard were applied to all participants with no potential for partial or differential verification biases. Furthermore, the reference standard was determined after serial standardised examinations by experienced team physicians reducing the risk of reference standard misclassification [32].

Conversely, there are a number of limitations which could challenge internal validity. Firstly, as a result of using routinely collected clinical data, there were inevitably missing data. However, the reported diagnostic accuracy metrics for the HIA01 off-field screening tool are consistent with a previously reported Rugby World Cup study with no missing data [6]. Excluded cases also had similar characteristics to included players. Taken together, this suggests that the findings are likely to be robust to selection bias.

Secondly, there is the possibility of diagnostic review and incorporation biases [32]. During normal sports medicine practice the team doctor predominantly conducts both off-field screens and subsequent diagnostic assessments. Team doctors may incorporate the screening test result in their overall diagnostic assessment or the knowledge of the screening test result might influence the interpretation of subsequent confirmatory evaluations. Both of these factors would be expected to improve the apparent accuracy metrics.

Thirdly, the diagnosis of concussion is inherently subjective and ill-defined, as acknowledged in the Berlin concussion consensus document [4]. Imperfect diagnosis of concussion could therefore lead to misleading accuracy metrics, with the extent of the systematic error dependent upon the frequency of misclassifications and the degree of correlation between index test and reference standard errors. Alternative approaches to a ‘fuzzy’ or imperfect gold standard include panel consensus diagnosis, latent class analysis, or reframing the research question from a diagnostic accuracy paradigm to the comparison of clinical outcomes with competing testing strategies [33]. Finally, further information bias could have arisen from players deliberately concealing symptoms, or underperforming on baseline assessments, to avoid missing games following a concussion diagnosis.

## Conclusions

The HIA process (immediate and permanent removal of players with overt signs or symptoms of concussion, plus off-field screening for head impact events with unclear circumstances or consequences) demonstrated good performance for the identification of concussion. Removal-from-play decisions using the off-field HIA01 screening tool had moderate sensitivity and good specificity for detecting concussion. Theoretical screening performance worsened if baseline or normative thresholds were strictly applied. These findings support expert recommendations that clinical judgement should be used in the assessment of athletes following head impact events. Tandem gait, 5-word immediate recall and Maddocks’s questions had low sensitivity and provided minimal or no diagnostic gain beyond symptoms, digits backwards, delayed recall and clinical signs for the identification of concussion cases. Substitution or alteration of these sub-tests with different or enhanced modes (e.g. 10 word immediate recall) could provide a target for improving screening tool performance.

## Data Availability

The datasets generated and analysed during the current study are not publicly available due to the terms of athlete consent and ethical approvals. However, anonymised data may be available from the corresponding author on reasonable request.

## Abbreviations

AUROC: Area under the receiver operating characteristic curve
CI: Confidence intervals
HIA: Head injury assessment
SCAT: Sports Concussion Assessment Tool
